# Prevalence of *Campylobacter hepaticus* specific antibodies among commercial free-range layers in Australia

**DOI:** 10.3389/fvets.2022.1058110

**Published:** 2022-11-14

**Authors:** Chithralekha Muralidharan, Jiongrui Huang, Arif Anwar, Peter C. Scott, Robert J. Moore, Thi Thu Hao Van

**Affiliations:** ^1^School of Science, RMIT University, Bundoora, VIC, Australia; ^2^Scolexia Pty Ltd., Moonee Ponds, VIC, Australia

**Keywords:** *Campylobacter hepaticus*, spotty liver disease, chicken, seroprevalence, ELISA, sera, immunoassay

## Abstract

Spotty liver disease (SLD) caused by *Campylobacter hepaticus* affects the health and productivity of layer hens and is a disease of concern in poultry. In this study, blood and cloacal swab samples were collected from 709 birds across 11 free-range layer farms from different regions of Australia. The prevalence of *C. hepaticus* specific antibodies and DNA was assessed using a *C. hepaticus* specific ELISA and PCR and its correlation with mortalities and changes in egg production was analyzed to better understand the seroprevalence of *C. hepaticus* in Australian free-range layer farms. *C. hepaticus* specific antibodies were detected from birds in four of the five farms that had no history of SLD with seroprevalence as high as 41% in one of the farms. Seroprevalence of anti-*C. hepaticus* antibodies among flocks that had an active or previous SLD outbreak varied between 2 and 64%. *C. hepaticus* DNA was detected from birds in three farms with no known SLD history and five farms with confirmed SLD outbreaks. A good correlation was observed between the ELISA and PCR results with a Pearson correlation coefficient value of 0.85 (*p*-value = 0.001). No correlation was observed between the flock size or flock age and ELISA or PCR outcomes, and no significant difference between the seroprevalence of anti-*C. hepaticus* antibodies among flocks with or without a known history of SLD was established (*p* = 0.143). This study demonstrates the usefulness of *C. hepaticus* specific ELISA and PCR in identifying the occurrence of mild or sub-clinical SLD and provides a broader and more complete understanding of SLD epidemiology that will inform future research aimed at the development of methods to control SLD, such as appropriate biosecurity measures, vaccines, and feed additives.

## Introduction

Spotty liver disease (SLD) emerged as a disease of concern in the UK and Australian egg farming sectors during the mid-1980s and is now, one of the most common infectious diseases in free-range layers (1–4). The consumer preference for free-range eggs and concomitant shift toward free range egg farming is considered as the main reason for increased SLD incidence in recent years (1, 3, 5). Variable production loss and mortalities among flocks at and around peak lay has been reported during SLD outbreaks, ranging from no notable drop in egg production to severe production drop of up to 25% and cumulative mortalities of up to 11% (1, 4–7). The causative agent of SLD was identified and named as *Campylobacter hepaticus* in 2016, several decades after the first reports of a disease with clinical manifestations similar to SLD, known as Avian vibrionic hepatitis, was documented (2, 8–12). Recently, another species of campylobacter named as *Campylobacter bilis* has also been associated with SLD (13). Since its characterization, *C. hepaticus* has been isolated and reported from layers in the UK, USA, Jordan, and New Zealand, and anecdotal reports indicate it is widespread throughout Europe and other regions, confirming the prevalence of *C. hepaticus* in geographically distinct locations around the world and emphasizing the global need for control measures to mitigate SLD (12, 14–17).

Feed additives such as isoquinoline alkaloids and biochar have been shown to provide some benefits in ameliorating SLD or reducing *C. hepaticus* carriage (18, 19). However, in-feed and water administration of antibiotics such as amoxycillin, linco-spectin, or chlortetracycline is still the most effective way to control SLD. However, antibiotic resistant field isolates of *C. hepaticus* harboring tetracycline resistance genes have already been identified, and that emphasizes the need for alternate treatment options and control measures (1, 20). Understanding the epidemiology of SLD and how birds respond to *C. hepaticus* infection is important in designing and applying appropriate biosecurity standards and for developing ways to control the disease.

The epidemiology of SLD had previously been investigated using *C. hepaticus* specific PCR and bacterial culturing methods. *C. hepaticus* DNA was detected in a variety of environmental sources such as soil, water, dust, insects, rodents, and wild birds, that indicated the likely transmission routes of *C. hepaticus* in poultry farms. However, viable *C. hepaticus* was not recovered from any of the environmental sources (21). Later, it was demonstrated that *C. hepaticus*, like other campylobacters can survive in a viable but non culturable state (VBNC). The long-term survival of *C. hepaticus* in the environment in a VNBC state has been hypothesized to play an important role in SLD epidemiology and *C. hepaticus* transmission (22).

*Campylobacter hepaticus* seroprevalence can be defined as the percentages of birds that have detectable levels of anti-*C. hepaticus* antibodies in their blood produced during a current or previous *C. hepaticus* infection. Investigation of seroprevalence of *C. hepaticus* in commercial free-range farms would further our understanding of the incidence of disease, both symptomatic and asymptomatic, by identifying the number of birds that have seroconverted. Such an enzyme linked immuno sorbent assay (ELISA)-based survey would be an important extension of the polymerase chain reaction (PCR) based molecular epidemiology work reported previously and could move beyond identifying birds with current infections, to identify birds that have previously been infected. This will give a much more complete view of the overall epidemiology of the disease.

Two ELISAs have been developed to detect *C. hepaticus* specific antibodies, namely SLD-ELISA1, based on *C. hepaticus* total proteins (23) and SLD-ELISA2, based on a recombinant immunogenic fragment of filamentous hemagglutinin adhesin (FHA) protein of *C. hepaticus* (paper submitted for publication). The recombinant protein fragment used in SLD-ELISA2 specifically detect anti-*C. hepaticus* antibodies in bird sera and was not cross-reactive with anti-*Campylobacter jejuni* or *Campylobacter coli* antibodies. SLD-ELISA1 showed 96% specificity and 98% sensitivity (23) whereas SLD-ELISA2 showed 95% specificity and 93% sensitivity (to be published elsewhere). Cross-reactivity of SLD-ELISA2 with *C. bilis* anti-FHA antibody is yet to be determined. SLD-ELISA2 was used to investigate the seroprevalence of *C. hepaticus* specific antibodies among hens in Australian free-range layer farms as it is more convenient to perform and standardizable than SLD-ELISA1. Over one thousand four hundred sera and cloacal swab samples were collected from 11 free-range layer farms, seven of which had a history of SLD, and assayed using SLD-ELISA2 and SLD specific PCR. The PCR amplifies glycerol kinase gene that is 100% specific for the bacteria causing SLD (*C. hepaticus* and *C. bilis*) and is highly sensitive with a detection limit of 7.9 cfu of *C. hepaticus*/ reaction (24). The ELISA and PCR results were analyzed alongside the egg production and mortality data provided by the farms to better understand the seroprevalence of *C. hepaticus* in Australian free-range layer farms and its correlation with mortalities and production drops. The outcomes of this study could inform future research aimed at the development of methods to control SLD, such as appropriate biosecurity measures, vaccines, and feed additives.

## Materials and methods

### Experimental design

A total of 709 blood and cloacal swab sample pairs were collected from 11 commercial free-range layer farms in Australia over a period of 9 months. Animal ethics approval was not required as the samples were collected by the veterinarians in the course of their routine farm visits for veterinary care and surveillance. Samples from each farm were collected in a single visit. Farms 1, 3, 4, 5, 7, 10 and 11 were in Victoria. Farm 2 was in New South Wales and Farms 6A, 6B, 8 and 9 were in Queensland. The age of the layer flocks at the time of sample collection ranged from 33 to 64 weeks in different farms and the number of birds in each flock ranged between 4,000 and 22,500. Blood and cloacal swab sample pairs were collected from 50 to 52 birds in each farm except for Farm 6. One hundred blood and cloacal swab sample pairs each were collected from flocks housed in two well separated sheds/ranges in Farm 6, one with an active SLD outbreak at the time of sample collection (referred as Farm 6A), and the other with no known history of SLD (referred as Farm 6B). The number of birds from which the blood and cloacal samples were collected in each farm is provided in column 1 of Table 1. Gross egg production and mortality data several weeks pre- and post-sample collection were obtained from farms to investigate the correlation between them, ELISA, and PCR results.

**Table 1:** Prevalence of anti-*Campylobacter hepaticus* antibodies and *C. hepaticus* DNA among Australian free-range layers.

**Farm ID (number of birds sampled)**	**Flock size (approx.)**	**Flock age at sampling** **(weeks)**	**Weeks before sample collection of the first (and recent) SLD bird death was reported**	**Percentage of birds tested positive in SLD-ELISA2**	***C. hepaticus*** **DNA detected in pooled (*n* = 10) cloacal swab samples by PCR**
**Farms with no known history of SLD**					
Farm 1 (50)	9,500	65	N/A	2	No
Farm 3 (51)	8,500	34	N/A	41	Yes (4/5)
Farm 5 (50)	20,000	35	N/A	2	Yes (1/5)
Farm 6B (100)	4,300	51	N/A	9	Yes (2/10)
Farm 10 (52)	9,000	52	N/A	0	No
**Farms with a known history of SLD**					
Farm 2 (52)	13,000	58	21 (15)	29	No
Farm 4 (50)	21,000	33	4	64	Yes (4/5)
Farm 6A (100)	4,000	64	41 (13)	11	Yes (3/10)
Farm 7 (52)	22,500	60	7	62	Yes (5/5)
Farm 8 (50)	20,000	35	16	4	Yes (1/5)
Farm 9 (50)	19,000	38	6	2	No
Farm 11 (52)	17,700	46	11	35	Yes (1/5)

NA, not applicable.

### Sample collection – blood, cloacal swabs, and bile

Blood and cloacal samples from individual birds were collected aseptically and transported to the laboratory within 48 h of collection, with ice packs to minimize protein and DNA deterioration. Blood samples in vacutainers (BD Diagnostics) were centrifuged at 2,000 × g for 15 min at 4°C to separate blood clots from the sera. The sera were transferred to Eppendorf tubes and stored at −20°C until used. The fecal material on cloacal swabs (sterile dry cotton swabs) were resuspended in 200 μl sterile phosphate buffer saline (PBS) solution and stored at −20°C until used. Bile samples were collected aseptically using syringe and needles from 4 to 7 birds that showed clinical SLD manifestations in Farm 2 and Farm 6A. They were plated on Brucella agar (BD) supplemented with 5% Horse blood (Equicell, Australia) and incubated at 37°C under microaerobic conditions provided by Campygen gas packs (Thermo Fisher Scientific) for 2–5 days. The egg production and mortality data was collected from the farms several weeks post sample collection.

### Polymerase chain reaction to detect *C. hepaticus* DNA

DNA was extracted from cloacal swab suspensions (10–12 samples from the same farm pooled together) using a DNeasy^®^ PowerSoil^®^ Pro Kit (Qiagen) as per the manufacturer's protocol. The extracted DNA samples were stored at −20°C until used. *C. hepaticus* specific end-point PCR was performed as described previously (24) with a few exceptions. Briefly, MyTaq™ Red Mix (2X) (Bioline/ Meridian Bioscience) was used in a final reaction volume of 20 μl including the forward and reverse primers (G2F3: CAGGAGTTTTACCACAATTC; G2R2: CAAGCTAAAACAGGTTTGG) at a final concentration of 250 nM each, and 5 μl of extracted DNA preparation. Amplification was carried out in an Eppendorf Mastercycler Pro thermocycler under the cycling conditions, 95°C for 1 min, 35 cycles of 95°C for 30 s; 56°C for 30 s and 72°C for 10 s, and a final extension at 72°C for 5 min. PCR products were run on agarose gels and visual images were captured using a Gel Doc XR System (Bio-Rad). PCR that amplifies bacterial 16S rRNA gene sequences was also performed to validate the quality of extracted DNA (2).

### SLD-ELISA2 to detect *C. hepaticus* specific antibodies

SLD-ELISA2 was performed as described previously (paper submitted for publication). Briefly, Nunc Maxisorp 96 well ELISA plate (Thermo Fisher Scientific) wells were coated with 50 μl per well of 0.5 μg/ml purified FHA_1, 628 − 1, 899_ protein in phosphate buffer saline (PBS). The plates were incubated for 2 h at room temperature (RT) and washed with 200 μl PBS containing 0.05% tween 20 (PBST). Non-specific binding sites were blocked using 200 μl of blocking solution (5% skim milk powder in PBS) and incubated overnight at 4°C. The wells were washed twice with PBST and 100 μl of chicken sera diluted a thousand-fold in blocking solution was added. The plates were incubated for 2 h at RT followed by four PBST washes. Goat anti-chicken IgY-HRP (Thermo Fisher Scientific) antibody diluted two thousand-fold in blocking solution was added and incubated for an hour at RT. After four PBST washes, 50 μl of Novex 3,3′,5,5′-tetramethylbenzidine chromogenic substrate (TMB, Invitrogen) was added and incubated for 15 min followed by the addition of 50 μl of 2 M sulphuric acid to stop the reaction. The absorbance was measured at 450 nm using a POLARstar Omega Plate Reader (BMG LABTECH).

### Statistical analysis

All statistical analysis was carried out using GraphPad Prism (9.3.1) software (San Diego, CA, USA). Correlation between the flock size, flock age, ELISA and PCR results were determined using multiple variable analysis correlation matrix by assuming Gaussian distribution of samples. The *p*-value was calculated at 95% confidence interval. Correlation between the sample collection time post first SLD outbreak and percentage of birds tested positive to SLD-ELISA2, and PCR was also calculated. The seroprevalence of anti-*C. hepaticus* antibodies among flocks with and without a known history of SLD were compared using the Mann–Whitney non-parametric *t*-test.

## Results

### Prevalence of *C. hepaticus* among Australian free-range layers

The presence and quality of extracted bacterial DNA in all samples was confirmed by 16S rRNA PCR. *Campylobacter hepaticus* DNA was detected in the cloacal swab samples in five of the seven farms (Farms 4, 6A, 7, 8, and 11) that had a current or previous SLD outbreak. Farms 4 and 7 had the highest proportion of birds that harbored *C. hepaticus* DNA. *C. hepaticus* DNA was also detected in three of the five farms (Farms 3, 5, and 6B) with no known history of SLD. The results are presented in Table 1 along with the details of flock size, age, SLD status and ELISA results. Bile samples were collected from Farms 2 and 6A, that had active SLD outbreaks, at the same time as blood and swab sample collection. *C. hepaticus* was isolated from bile samples from Farm 6A but not from Farm 2. Flocks in Farm 2 were being treated with antibiotics at the time of sampling whereas flocks in Farm 6A were not on any medication as the farm was managed under organic farming practices.

### Prevalence of anti-*C. hepaticus* antibodies among Australian free-range layers

Anti-*C. hepaticus* antibodies were detected in birds from all seven farms with a known history of clinical SLD (Table 1). Correlating with the PCR results, farms 4 and 7 had the highest number of birds, 64 and 62% respectively, seropositive to *C. hepaticus*. SLD-ELISA2 also identified birds exposed to *C. hepaticus* earlier in their life, that had cleared the bacteria in Farms 2, and 9. *C. hepaticus* DNA was not detected from birds in these farms. Anti-*C. hepaticus* antibodies were also detected in birds from four of five farms with no known history of SLD. Among them, Farm 3 had the highest number of birds (41%) that had seroconverted.

### Correlation between egg production, mortality, presence of *C. hepaticus* DNA and *C. hepaticus* immune status

**Figure 1:**
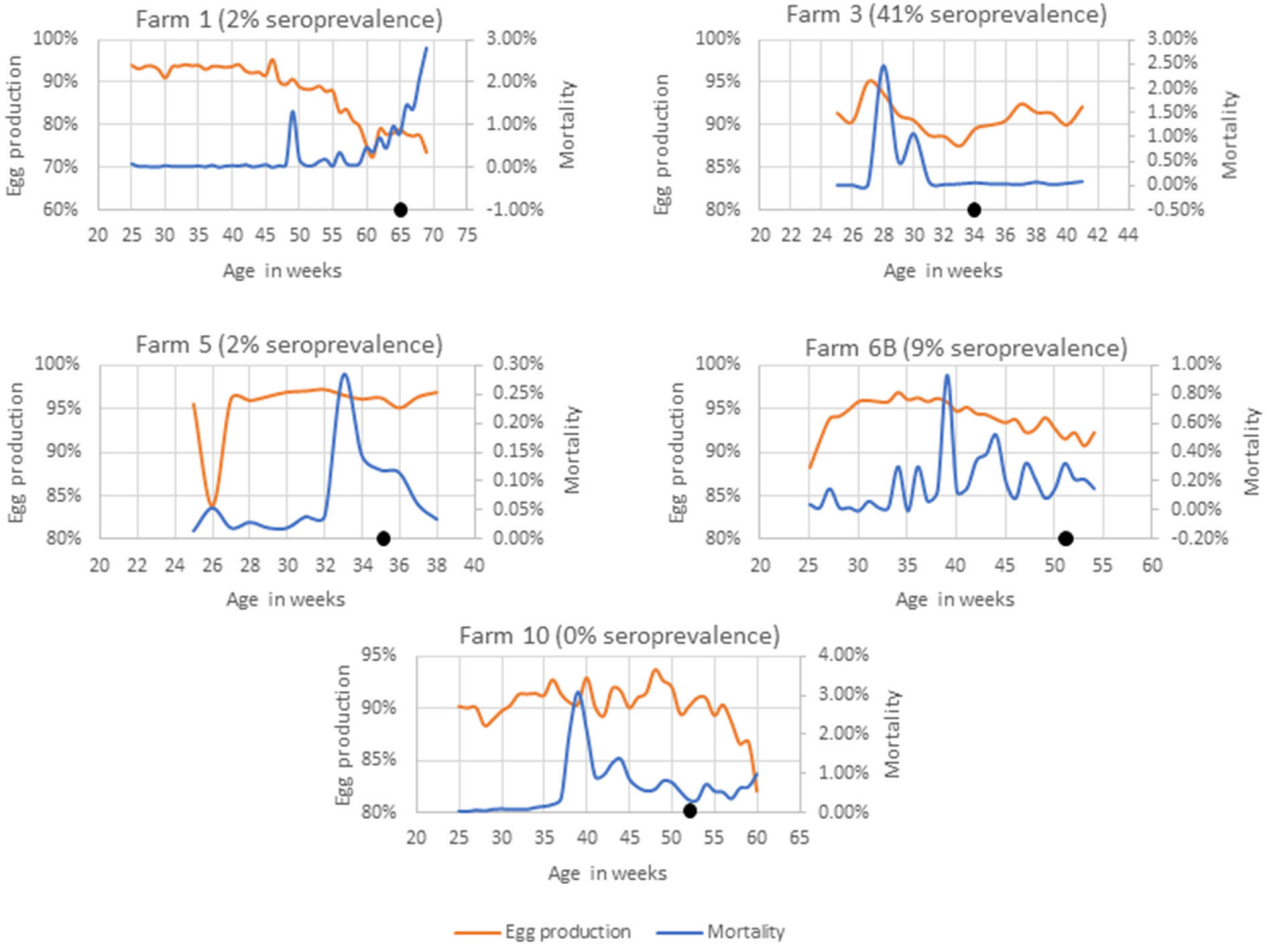
Mortality and egg production in farms with no known history of SLD. The black dot indicates the week of sample collection.

Farms 1, 3, 5, 6B, and 10 had no known history of SLD outbreaks (Figure 1). In farm 3, a sudden rise in weekly mortalities of up to 2.4% was observed 7 weeks prior to sample collection that was associated to a smothering event. The weekly egg production also dropped from 94% to 88%. Although the farm had no record of SLD, 41% of the birds had anti-*C. hepaticus* antibodies in their blood and four of the five pooled cloacal swabs had *C. hepaticus* DNA suggesting an SLD outbreak that was not identified in the routine veterinary surveillance.

In Farms 1 and 5, 2% of birds (a single bird) were seropositive to *C. hepaticus. C. hepaticus* DNA was also detected in one of the five pooled cloacal swab samples from Farm 5. However, there was no notable production drop or mortalities in Farm 5. Farm 1 had a confirmed *Erysipelothrix rhusiopathiae* outbreak 5 weeks prior to sample collection and that could be the reason for the production drop and mortalities in the later weeks.

In Farm 6B, 9% of the birds were seropositive to *C. hepaticus* and two of the 10 pooled cloacal swabs had *C. hepaticus* DNA. Mortalities peaked at 0.93% in week 39. However, no significant drop in egg production was noted. The birds in Farm 10 were *C. hepaticus* negative in SLD-ELISA2 and PCR. The farm had high mortalities during weeks 38–44 and a drop in egg production from week 58 that was associated with injurious pecking and behavioral issues frequently reported within the flock.

**Figure 2:**
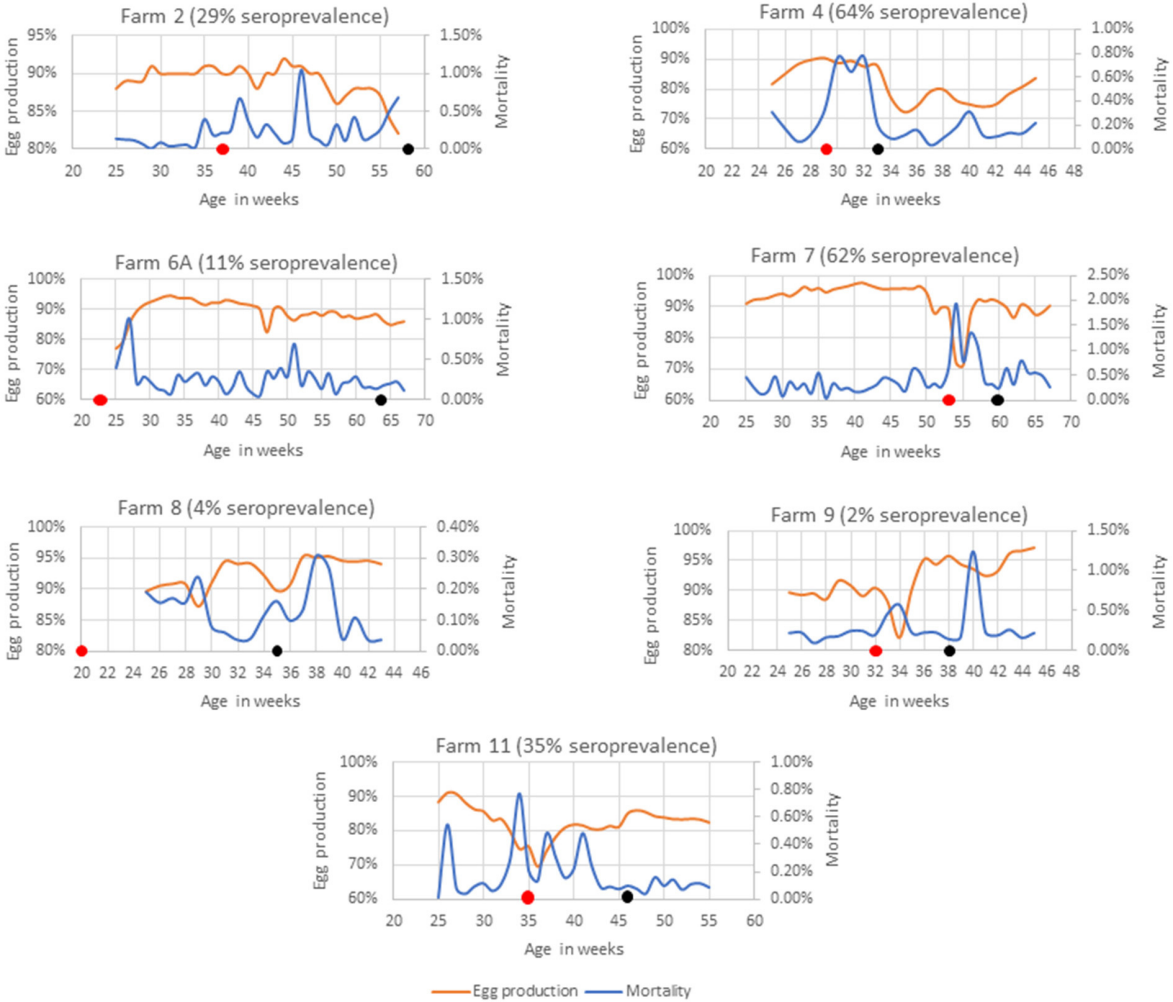
Mortality and egg production in farms with a known history of SLD outbreak. The black dot indicates the week of sample collection and the red dot indicates the week in which, the first SLD associated death was recorded.

Farms 2, 4, 6A, 7, 8, 9, and 11 had SLD outbreaks 4–41 weeks prior to sample collection (Figure 2). SLD associated bird mortalities were recorded 21 weeks prior to sample collection in Farm 2 and anti-*C. hepaticus* antibodies were detected in 29% of birds. However, no *C. hepaticus* DNA was detected. Weekly mortalities reached up to 1% in week 46 and weekly egg production dropped to 82% during the week of sample collection. In Farm 4, SLD outbreak occurred 4 weeks prior to sample collection and the birds were on water and in-feed antibiotic medication. An increase in mortality of up to 0.8% and up to 18% drop in weekly egg production was observed that correlated with the detection of anti-*C. hepaticus* antibodies in 64% of the birds and *C. hepaticus* DNA in four of the five pooled cloacal swab samples.

Spotty liver disease associated bird deaths were reported in Farm 6A since the birds were 22 weeks of age. The birds were not on any antibiotic treatment as they were managed under organic farming practices and the flock experienced occasional SLD outbreaks. The latest SLD death was documented 13 weeks prior to sample collection. The mortalities were generally low and egg production was mostly above 85%. Only 11% of birds were positive in SLD-ELISA2. However, three of the five pooled cloacal swabs had detectable levels of *C. hepaticus* DNA. Furthermore, *C. hepaticus* was isolated from bile culture and confirmed active SLD infections in this flock.

Farm 7 had high mortalities from week 15, as early as the birds were brought from rearing to the layer farm. A clinical SLD outbreak was reported when the flock was around 53 weeks of age. A steep rise in mortalities of up to 2% in week 54 coincided with a drop in egg production to 71% that lasted for a period of 6 weeks that correlated well with the detection of *C. hepaticus* DNA in all five pooled cloacal swab samples and anti-*C. hepaticus* antibodies in 62% birds.

SLD was confirmed in Farm 8 when the flock was 19 weeks of age. However, only 4% of the birds had anti-*C. hepaticus* antibodies and one of the five pooled cloacal swab samples had *C. hepaticus* DNA. Notably, the farm had very low mortalities. The egg production dropped to 87% only for a period of 2 weeks and was above 90% in the remaining weeks.

Only one bird out of 50 birds tested positive to SLD-ELISA2 in Farm 9 and *C. hepaticus* DNA was not detected in cloacal swabs. SLD outbreak was noticed 6 weeks prior to sample collection, marked by a sudden drop in egg production to 82%. The egg production recovered to a normal rate within 2 weeks.

A sustained egg production drop throughout the lay, down to as low as 69%, and mortalities per day of up to 0.8% was observed in Farm 11. *C. hepaticus* DNA was detected in one of five pooled cloacal swab samples and 35% birds were seropositive to *C. hepaticus*.

### Statistical analysis

**Figure 3:**
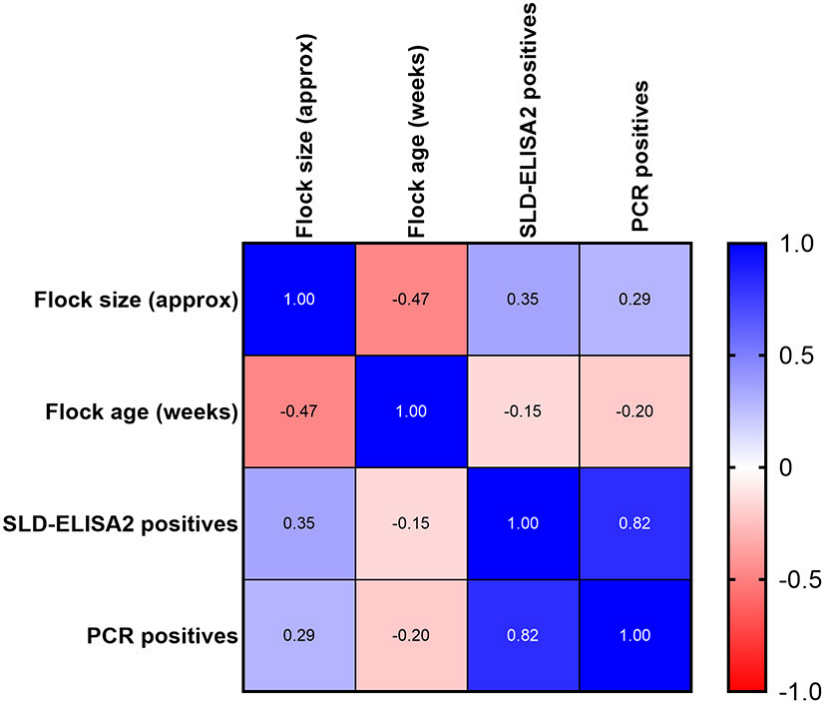
Pearson correlation, *r* between flock size, flock age, and percentage of birds positive to SLD-ELISA2 and PCR.

A good correlation was observed between SLD-ELISA2 and PCR results with a Pearson correlation coefficient, *r* value of 0.85 and a *p*-value of 0.001. The confidence interval of *r* value was 0.5371–0.9568. As expected, no correlation was observed between the flock size or flock age on ELISA and PCR outcomes (Figure 3). Although, the majority of seroconverted birds were in flocks with a known history of SLD, a statistically significant difference between the seroprevalence of anti-*C. hepaticus* antibodies among flocks with or without a known history of SLD could not be established (*p*-value = 0.143; Table 2). Similarly, no significant difference was observed between the sample collection time post first SLD outbreak and the ELISA (*p*-value = 0.307) or PCR (*p*-value = 0.471) outcomes.

**Table 2:** Seroprevalence of *Campylobacter hepaticus* in flocks with/without a known history of SLD.

	**Farm/shed (*n*)**	**Sample size (*n*)**	**ELISA positive (*n*)**	**Seroprevalence (mean)**	**Seroprevalence (median)**	***P*** **-value**
Flocks with a known history of SLD	7	406	111	27%	29%	0.1427
Flocks with no known history of SLD	5	303	32	11%	2%	
Total	12	709	143	20%		

## Discussion

An important finding of this study was the identification of seroconverted birds in four out of five farms that had no previous history of SLD. In Farm 3, 41% of the birds had anti-*C. hepaticus* antibodies and four of the five pooled cloacal swabs detected *C. hepaticus* DNA. The farm had records of a smothering event 7 weeks prior to sample collection that resulted in 2.4% mortality, followed by a production drop of up to 5% that lasted for 5 weeks. Smothering events are common in cage-free flocks that can result in death of a few to several hundred birds in a single event. The trigger for this piling and suffocating to death behavior, categorized as panic smothers, nest box smothers, and recurring smothers are unclear in most instances (25, 26). The occurrence of a smothering event around the same time as the production drop may have masked an actual SLD outbreak in this flock, evidenced by a production drop 2 weeks post smothering event and further confirmed by the positive results to SLD-ELISA2 and PCR. It also possible that the smothering event might have acted as a predisposing factor for the SLD outbreak. The predisposing factors associated with clinical SLD that result in high mortalities and production loss include disruptions to bird husbandry such as availability of feed and water, birds accessing stagnant water in the range, hot-humid weather, wet litter, overcrowding in shelter houses on the range, inadequate feed space, cannibalism, round worm or coccidial damage to intestinal lining along with the hormonal changes, and stress in birds during lay (1, 5).

*Campylobacter hepaticus* can infect layers in rearing without showing any clinical signs (5, 21). Identification of seroconverted birds in Farms 1, and 5, with no known history of SLD suggests that pullets might have been exposed to *C. hepaticus* during rearing and cleared the infection prior to the onset of lay. A drop in egg production and increase in mortalities among layer hens are associated with SLD or classified as clinical SLD only if the bird necropsies demonstrate characteristic gray or cream spots on the liver, which is the hallmark of SLD. This explains the reason for mild *C. hepaticus* infections (no obvious signs of SLD) or sub-clinical SLD (a drop in egg production and increase in mortalities was noticed but no spots in the liver was identified to confirm SLD) to go unnoticed in farms. The present study demonstrates the usefulness of ELISA and PCR testing to determine the incidence of mild or sub-clinical SLD by identifying seroconverted birds in farms with no known history of SLD.

Interesting differences in antibody responses were observed among birds in different farms with a known history of SLD based on the location of the farm and recovery period from the latest SLD exposure. Seroprevalence of anti-*C. hepaticus* antibodies were higher in Farms 4 (64%) and Farm 7 (62%) that had SLD outbreaks 4 and 7 weeks respectively, prior to sample collection. Whereas Farms 2, 6A, 8 and 11 had SLD exposures 11–15 weeks prior to sample collection and had a lower proportion of birds (4%−35%) with detectable levels of anti-*C. hepaticus* antibodies. Prevalence of *C. hepaticus* DNA also demonstrated the same trend. It is likely that the antibody levels had dropped over time in many birds within these flocks that resulted in low seropositivity. The egg production in Farms 2, 4, and 11 did not recover to the standard/expected production levels corresponding to the flock age after SLD outbreak whereas the production in Farms 6A, 7, 8, and 9 recovered to the standard production levels.

Farms 6A, 6B, 8 and 9 were in Queensland and all except Farm 6B had previous histories of SLD outbreaks. SLD outbreaks were detected in the farms 6A, 8 and 9 at 13, 16, and 6 weeks before sample collection. However, only 2%−11% of birds had detectable levels of anti-*C. hepaticus* antibodies. Detection of seropositivity in 9% of the birds in Farm 6B with no known SLD history indicated the likely transmission of SLD within Farm 6 between flocks maintained in different sheds/ranges. It is also possible that both the flocks might have been infected with the bacteria during rearing and birds in shed 6A broke with SLD outbreak earlier than shed 6B. Another interesting observation is that the egg production drop and mortalities in all these farms were comparatively mild, concurrent with the low prevalence of *C. hepaticus* DNA and anti-*C. hepaticus* antibodies in flocks. This is suggestive of the variability in virulence of *C. hepaticus* strains. When assessing the degree of pathogenicity of different *C. hepaticus* strains, we found that the QLD19L isolated form Queensland was less pathogenic than the NSW44L strain isolated from New South Wales (27). Although SLD specific PCR can detect both *C. hepaticus* and *C. bilis*, whether SLD-ELISA2 can detect anti-FHA antibodies produced from *C. bilis* infection needs to be experimentally determined. The lower mortalities and *C. hepaticus* transmission may be also due to different biosecurity measures enforced by different farms or due to differences in the presence of other currently uncharacterised/unrecognized predisposing factors. Earlier, flocks with more than 12,000 birds were considered at risk of SLD (28). However, no such correlation between SLD incidence and flock size was established in the current study. In addition, no statistical correlation was found between the flock age and SLD incidence as four farms included in this study had SLD outbreaks during peak lay, two farms reported SLD outbreak in their early lay and one farm broke with SLD in late lay.

The observations from this study support the hypothesis that *C. hepaticus* infection, by itself, is not sufficient to produce clinical SLD; demonstrated by the presence of seroconverted birds in farms with no previous history of SLD. Other predisposing factors need to be present before clinical SLD manifests. An extensive immunological and molecular survey of birds in early lay can provide information on whether the birds are exposed to *C. hepaticus* in rearing or early lay and whether exposure in rearing provides any level of protection in the future. Even though detectable levels of antibodies drop over time, memory immune cells circulating in the blood stream may be sufficient to provide protection against future infections, which is indicative of the likelihood of SLD vaccine efficacy.

## Data availability statement

The original contributions presented in the study are included in the article/supplementary material, further inquiries can be directed to the corresponding author/s.

## Ethics statement

Animal ethics approval was not required as the samples were collected by the veterinarians in the course of their routine farm visits for veterinary care and surveillance.

## Author contributions

TV and RM conceived the study and edited the manuscript. CM designed and performed the experiments and drafted the manuscript. JH, AA, and PS collected the samples and arranged for sample collection by other veterinarians. All authors have edited and approved the final manuscript.

## Conflict of interest

Authors JH, AA, and PS were employed by Scolexia Pty Ltd. The remaining authors declare that the research was conducted in the absence of any commercial or financial relationships that could be construed as a potential conflict of interest.

## Publisher's note

All claims expressed in this article are solely those of the authors and do not necessarily represent those of their affiliated organizations, or those of the publisher, the editors and the reviewers. Any product that may be evaluated in this article, or claim that may be made by its manufacturer, is not guaranteed or endorsed by the publisher.
